# Supplementary material for: Mutation of an Essential 60S Ribosome Assembly Factor MIDASIN 1 Induces Early Flowering in Arabidopsis
Source: Int J Mol Sci. 2022 Jun 10;23(12):6509. doi: 10.3390/ijms23126509 (PMC9223865; doi:10.3390/ijms23126509)
Supplement: Supplementary file 1 [file ijms-23-06509-s001.zip › ijms-1726612-supplementary.pdf]

Mutation of an Essential 60S Ribosome Assembly Factor, *MIDASIN 1*, Induces Early Flowering in *Arabidopsis*

Ke Li [1], Pengfei Wang [2], Tingting Ding [2], Lei Hou [1], Guanghui Li[1], Chuanzhi Zhao[1], Shuzhen Zhao[1], Xingjun Wang [1], Pengcheng Li [1*]

[*] Corresponding authors: P.L. (email: lpcsaas@outlook.com)

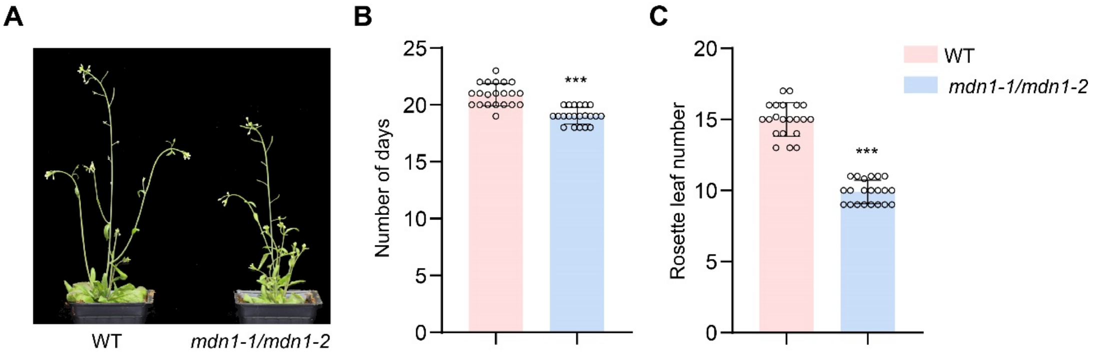

Figure S1. *mdn1-1/mdn1-2* is early flowering. (A) WT and *mdn1-1/mdn1-2* plants are grown on long days condition. Scale bars, 2 cm. (B-C) The number of days and rosette leaf number at flowering grown in LD. Values are mean ±SE (n=20, ⋆⋆⋆P< 0.001, Student's t-test).

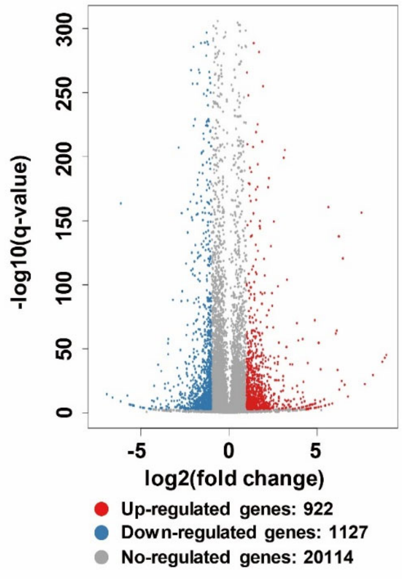

Figure S2. Volcano plots of fold changes. Log_2 of fold-change and $-log_{10}$ of the q-value are present on the axis. Red, blue and gray dots represent significantly up-, down-regulated and no-regulated genes ($|Log_2$ (fold-change) $| \geq 1$, q-value < 0.001).

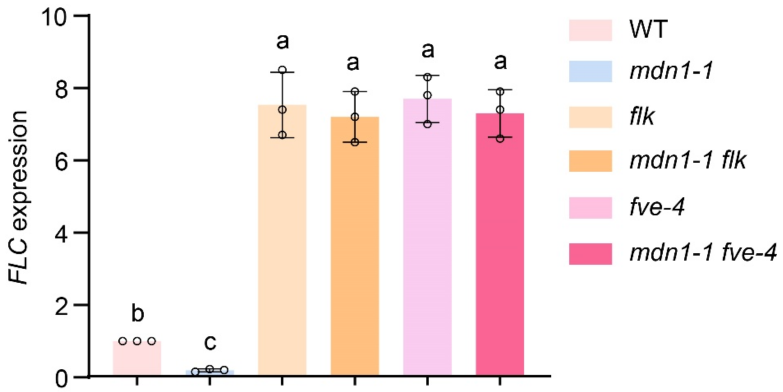

Figure S3. *FLC* expression in *flk mdn1-1* and *fve-4 mdn1-1*mutants. qRT-PCR results show relative expression levels of *FLC* in 5 DAG WT, *mdn1-1*, *flk*, *fve-4*, *mdn1-1 flk* and *mdn1 fve-4* seedings. Values are mean ±SE. The relative expression of the indicated genes was normalized to the level of the *Actin2*.

Table S1. List of characterized ribosome assembly factors in Arabidopsis

Name	Function	AGI	Flowering	Reference
RH7	DEAD/DEAH box RNA helicase	AT5G62190	delay	[58]
MTR4	DEAD-box RNA helicase	AT1G59760	delay	[48]
IRP5	RNA binding	AT1G29250	delay	[54]
IRP6/RH14	DEAD/DEAH box RNA helicase	AT3G01540	delay	[54]
IRP7	RNA binding	AT2G34160	delay	[54]
IRP8/ALY3	RNA binding	AT1G66260	delay	[54]
IRP9	Polyadenylation	AT4G25550	delay	[54]
RRP7	RNA binding	AT5G38720	delay	[59]
LSG1-1/LSG1-2	GTPase	AT2G27200/ AT1G08410	delay	[56]
NUC2	Nucleolin proteins	AT1G48920	delay	[60]
XRN3	5'-3'exonucleases	AT1G75660	delay	[61]
APUM23	RNA binding	AT1G72320	delay	[62]
RPL27aC	Ribosomal protein	AT1G23290	delay	[63]
RPS5A	Ribosomal protein	AT3G11940	delay	[44]
RPL4A	Ribosomal protein	AT3G09630	delay	[64]

Table S2. Primers used in the study.

Primer name	Sequence (5’-3’)
mdn1-1 F	GAAATTGAATATAGAATCCAAAAATATTG
mdn1-1 R	ATCTTTTGGAGCCAATTATTTCCCTAGC
LBb1.3	ATTTTGCCGATTTCGGAAC
mdn1-2 F	TCTGAGAGAAAAATGCTTCGC
mdn1-2 R	TGTGGAAAGGCAACAGAATTC
flc-3 F	CGAGAAAAGGAAAAAAAAAAA
flc-3 R	CTGTTTCCCATATCGATCAAGGAT
flk-4 LP	TTTTAAGATGCCATAGCAGCC
flk-4 LP	TATTCCGTATGCTGGTTCCTG
fve-4 F	TTCAATGAAGAAGCACGTTCT
fve-4 R	TCATTCTTATCAGTACCTTCA
co LP	AAGCTGTTGTGACACATGCTG
co RP	CCCCTTCTTTCAGATACCAGC
ft-10 LP	GGTGGAGAAGACCTCAGGAAC
ft-10 LP	TTTTGGGAGACAAATTGATGC
clf-28 LP	TTCGGTTGGCACTAAACTCAC
clf-28 RP	TGTAGAAGATGGACCTGCCAG
qFLC F	TGAGAACAAAAGTAGCCGACAAG
qFLC R	GGCGGAGACGACGAGAAG
qFT F	CCAAGTCCTAGCAACCCTCA
qFT R	TACACTGTTTGCCTGCCAAG
qABI5 F	TCGACAAGGCTCTTTGACAC
qABI5 R	ATTACCGCTACCACCACCTC

qFLK F	GGAACTGGCTCACAAGTCCAA
qFLK R	CCATGAAGTTCTGAATAAGCTGCA
qFVE F	CAGTGGAAAGGTCTCGTCCC
qFVE R	GCAGCAACCCTTGGCTTAAC
qLD F	CGGTTTGCGTATTGGTCTTCG
qLD R	ACGACGACATCTTGGAGCTG
Actin-2 F	TTGACTACGAGCAGGAGATGG
Actin-2 R	ACAAACGAGGGCTGGAACAAG
